# OP64 Risk-Based Prioritization In Patients Referred For Transcatheter Aortic Valve Implantation: A Simulation Study

**DOI:** 10.1017/S0266462324001247

**Published:** 2025-01-07

**Authors:** Rafael Miranda, Peter Austin, David Naimark, Harindra Wijeysundera

## Abstract

**Introduction:**

Demand for transcatheter aortic valve implantation (TAVI) has increased in the last decade and has outpaced system capacity, impacting wait times and bringing undesirable health outcomes such as waitlist mortality and number of urgent procedures. Risk-based prioritization can improve equitable access to patients. In this study, we assess the impacts of different classifications and wait times for each risk group on health outcomes.

**Methods:**

We developed decision-analytic models that simulate the patient trajectory from referral to completion of TAVI. Using prediction models that can classify patients based on their risk of adverse events on the waitlist, we assessed the impacts of (i) the number of risk groups, (ii) size of the risk groups, and (iii) recommended wait times for each risk group, on waitlist mortality, hospitalization, and the proportion of urgent TAVIs. All scenarios were modeled under the same resource constraints, allowing us to explore the trade-offs between faster access to prioritized patients and deferred access to nonprioritized groups.

**Results:**

Increasing the number of risk groups from two to three, increasing the sizes of the higher-risk groups from five percent to 30 percent of the cohort each, and providing faster access to the higher-risk groups (five to three weeks for high-risk and 11 to five weeks for medium-risk) achieved the greatest reductions in mortality, hospitalizations, and urgent TAVIs (relative reductions of up to 29%, 23%, and 38%, respectively). However, this occurs at the expense of excessive wait times in the nonprioritized group (up to 25 weeks). The reduction in adverse events was lower when the nonprioritized group had more reasonable wait times.

**Conclusions:**

When developing and implementing waitlist prioritization strategies, it is important to consider the resource constraints of the system and the patient profile, as the benefits of providing faster access to prioritized patients can lead to unreasonable wait times for nonprioritized ones. In settings with long wait times, prioritization initiatives must be followed by expansion of supply to achieve optimal improvements in health outcomes.

